# Knowledge of neonatal danger signs among mothers attending well baby clinic in Nakuru Central District, Kenya: cross sectional descriptive study

**DOI:** 10.1186/s13104-016-2272-3

**Published:** 2016-10-25

**Authors:** Elizabeth Gathoni Kibaru, Amos Magembe Otara

**Affiliations:** 1 Department of Paediatric and Child Heath, Faculty of Health Sciences, Egerton University, Nakuru, Kenya; 2 Department of Reproductive Health, Faculty of Health Sciences, Egerton University, Nakuru, Kenya

**Keywords:** Danger signs, MCH booklet

## Abstract

**Background:**

Neonatal mortality has remained high in Kenya despite various efforts being applied to reduce this negative trend. Early detection of neonatal illness is an important step towards improving new born survival. Toward this end there is need for the mothers to be able to identify signs in neonates that signifies severe neonatal illnesses. The objective of the study was to determine the level of knowledge of mothers attending well baby clinics on postnatal neonatal danger signs and determine the associated factors.

**Study design:**

Cross sectional descriptive study.

**Study methods:**

Purposive sampling of Health care facilities that provide antenatal, delivery and postnatal services were identified. In each of the selected health facility structured questionnaires were administered to mothers with children aged six weeks to nine months attending well baby clinics. Frequencies, Chi square and multivariate logistic regression were determined using the SPSS software (version 20).

**Results:**

During the period of study 414 mothers attending well baby clinics were interviewed. Information on neonatal dangers was not provided to 237 (57.2%) of the postnatal mothers during their antenatal clinic attendance by the health care providers. Majority of mothers 350 (84.5%) identified less than three neonatal danger signs. Hotness of the body (fever) was the commonly recognized danger sign by 310 (74.9%) postnatal mothers. Out of 414 mothers 193 (46.6%), 166 (40.1%), 146 (35.3%) and 24 (5.8%) identified difficulty in breathing, poor sucking, jaundice and lethargy/unconsciousness as new born danger signs respectively. Only 46 (11.1%) and 40 (9.7%) identified convulsion and hypothermia as new born danger signs respectively. Education Level, PNC accompaniment by Spouse, Danger signs information to Mother, Explanation of MCH booklet by Care provider during ANC and Mother read MCH Booklet were factors positively associated with improved knowledge of neonatal danger sign. In multivariate logistic regression none of the factors tested were statistically significant in relation to level of knowledge.

**Conclusion:**

Knowledge of neonatal danger signs was low among mothers attending well baby clinic despite the information being available in the MCH booklets provided to the mothers during antenatal clinics.

## Background

Globally, almost three-quarters of neonatal deaths occur within the first seven days of delivery [1]. Million new-borns die during the first 4 weeks of life each year and world-wide neonatal mortality makes up 40% of the total child mortality [1, 2]. In a study by Matendo et al. [3] in Congo, they found that most neonatal deaths occurred soon after birth, and nearly three-quarters were caused by low birth weight/prematurity or asphyxia. In Kenya the neonatal mortality rate was reported in the 2014 demographic survey as 22 deaths per 1000 live births [4]. The high mortality and morbidity rates have been attributed to a significant break in the continuum of care in the service-delivery strategy after delivery. Care during Post Natal Clinic is critical for both the mother and baby [5]. In Kenya post natal care follow up remain very low which is comparable to studies elsewhere. Postnatal period has been identified as one of the periods when information concerning neonatal danger signs is passed on to the mother and this assist the mothers to identify children at risk and seek medical assistance early. In the Bangladesh demographic health survey showed that less than one in five newborns is checked by a health professional within six weeks of delivery with only 12% of babies receiving postnatal check-up by a trained health provider within the first two days of delivery [6]. In Kenya the percentage of mothers who attended postnatal care clinic within the first two days after delivery was 51% with no mention of percentage of new-borns who were reviewed within the same period [4]. The primary strategic of reducing mortality is to increase sustainable key health-practices and the use of essential services in communities. The most effective strategies to reduce mortality are those that treat the causes of early mortality. Various factors influence the women ability to seek care for their neonates. It has been noted that women’s utilization of maternal and neonatal health services are often influenced by perceived socio-cultural, economic and health system factors operating at the community, household and individual level as well as within the larger social and political environments and health care infrastructure [7, 8]. 

Early identification of new born danger signs by caregivers with prompt and appropriate referral serves as backbone of the programs aiming at reduction in neonatal mortality [9]. Neonates are more prone to show subtle signs of illness and these can only be identified by the immediate care givers who have adequate knowledge on features to look for. Listlessness or difficulty feeding are sometimes the only signs present and illness may advance quickly [10, 11]. Different tools to facilitate identification of these health problems and reduce neonatal mortality have been introduced into health programs in several countries. Integrated Management of Newborn and Childhood Illness (IMNCI) developed by the World Health Organization (WHO) focuses on assessment of general danger signs in the examination of children presenting with illness at health care centres. WHO in 2013 strongly recommended specific danger signs that should be assessed during each postnatal care contact and the new born should be referred for further evaluation if any of the signs are present [12]. The family should also be encouraged to seek health care early if they identify any danger signs in-between postnatal care visits [12]. The danger signs are as follows; stopped feeding well, History of convulsions, fast breathing (breathing rate >60/min) severe chest in-drawing, no spontaneous movement, fever (temperature >37.5 °C), low body temperature (temperature <35.5 °C), any jaundice in first 24 h of life, or yellow palms and soles at any age [12]. In a Multicentre study by Young Infants Clinical Signs Study Group it was noted that assessment of danger signs resulted in a high overall sensitivity and specificity for predicting the need for hospitalization of a new born in the first week of life [13].

In Kenya, the ministry of health has integrated mother and child information in one booklet which is provided to the mother during antenatal clinic. Information on danger signs have been in cooperated in the book for the care provider to advice the mothers and also for the mothers to read. Various studies in developing countries have demonstrated that despite availability of information on neonatal danger signs on MCH booklet maternal knowledge on the same remain very low [14, 15]. The study was therefore aimed at determining the maternal knowledge of neonatal danger signs in Nakuru central district in Kenya.

## Objectives

To determine the maternal knowledge of neonatal danger signs among postnatal mothers attending well baby clinic.

To determine the correlates of maternal knowledge of neonatal danger signs and the mothers’ demographic characteristics.

## Results

### Social demographic characteristics of the mothers who attended well baby clinic

Majority of the mothers who were interviewed were of reproductive age 18–35 years and 87.4% of them were married with 37.4% being first time mothers as shown in Table 1. Less than half (42.5%) of the mothers were accompanied by their spouse to the antenatal care clinic whereas only 29.5% were accompanied by their spouses to the postnatal care clinic. A minority (5.0%) of the postnatal mothers had not attained formal education and most of the mothers had access to a mobile telephone and the telephone set was personal in 93% of the mothers.

**Table 1:** Socio-demographic characteristics of mothers attending well baby clinic at Nakuru Central District (N = 414)

Characteristics	N	%
Age of the mother (years)		
Less than 18	6	1.4
18–35	360	87.0
36–45	48	11.6
Marital status		
Single	47	11.4
Married	362	87.4
Divorced/separated	5	1.2
Parity		
1	155	37.4
2–4	238	57.5
5+	21	5.1
Accompanied by spouse to ANC	176	42.5
Level of education		
None	21	5.0
Primary school	134	32.0
Secondary school	136	33.0
Middle level college	87	21.0
University	36	9.0
Access to mobile telephone	385	93.0
Ownership of accessible mobile telephone		
Personal	384	93.0
Family	12	3.0
Others	18	4.0

*ANC* antenatal clinic

### First postnatal clinic attendance

All mothers who were interviewed during the study period reported to have attended postnatal clinic with over 50% visiting the hospital within 1–2 week after delivery as shown in Table 2. Majority (70.5%) of the postnatal mothers were not accompanied by their partners.

**Table 2:** First postnatal review by mothers attending well baby clinic at Nakuru Central District (N = 414)

Attribute	N	%
Within 48 h	14	3.4
1–2 weeks	209	50.9
4–6 weeks	188	45.7
Accompanied by spouse to PNC	122	29.5

*PNC* post natal clinic

### Access and utilization of MCH booklet

The study showed that almost all mothers who came to the study sites for well-baby clinic had been provided with the standard MCH booklet (98.3%) during the ANC clinic as shown in Table 3. However, only 61% of them were explained the contents of the booklet by the health care providers and only 42.5% were informed about neonatal danger signs. Among the postnatal mothers up to 28.5% of them did not read the instructions in their MCH booklet.

**Table 3:** MCH booklet availability and utilization for mothers attending MCH clinic at Nakuru Central District (N = 414)

Mother attributes	N	%
MCH booklet during ANC	406	98.3
Explanation received on content of MCH booklet from the care provider during ANC	252	61
Received information on neonatal danger signs from care provider during ANC	177	42.8
Read all the instructions in the MCH booklet during ANC	296	71.5

### Maternal knowledge on neonatal danger signs

**Fig. 1:**
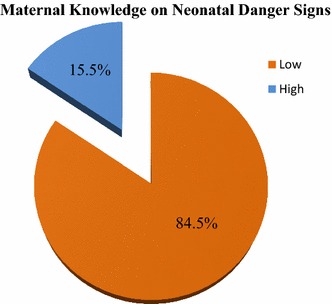
Maternal level of knowledge on neonatal danger signs

The scoring of neonatal danger signs was evaluated and scored. For mothers who were able to identify less than three were classified as having low knowledge and those who scored more than three were classified as having good knowledge of neonatal danger signs. Majority of mothers 350 (84.5%) had low level of knowledge as shown in Fig. 1 (figure attached as a supplement).

### Recognition of neonatal danger signs

**Fig. 2:**
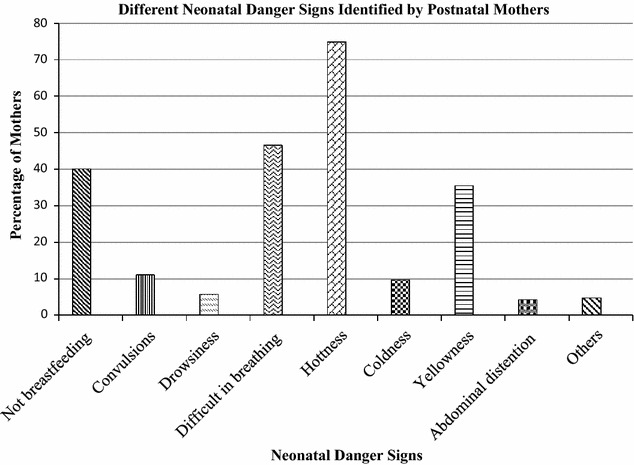
Different neonatal danger signs identified by postnatal mothers

Hotness of the body (fever) was the commonly recognized danger sign by 310 (74.9%) postnatal mothers. Out of 414 mothers 193 (46.6%), 166 (40.1%) and 24 (5.8%) identified difficulty in breathing, poor sucking and lethargy/unconsciousness as new born danger signs respectively. Only 46 (11.1%) and 40 (9.7%) of mothers identified convulsion and hypothermia as new born danger signs respectively. Figure 2 shows percentage of mothers who were able to identify different neonatal danger signs (attached as a supplement).

### Factors associated with maternal knowledge of neonatal danger signs

#### Inability to breastfeed as a neonatal danger signs

The mother’s having been accompanied to postnatal clinic by their partners, having read the MCH booklet, receiving information from health care workers on neonatal danger signs and parity had positive influence on maternal knowledge of inability to breastfeed as a neonatal danger sign and this was significant with a P value of 0.008, 0.000, 0.000 and, 0.013 respectively as shown in Table 4. Mothers marital status, age of the mother, level of education and being explained the content of MCH booklet by the care provider were not significant in respect to the maternal knowledge of inability to breastfeed of neonatal danger signs.

**Table 4:** Factors associated with maternal knowledge of neonatal danger signs

Attributes	Body hotness	Breathing difficulty	Convulsion	Drowsiness	Breastfeeding inability	Cold extremities	Abdominal distension	Jaundice
Age	0.340	0.010	0.055	0.615	0.359	0.487	0.611	0.613
Marital status	0.217	0.191	0.032	0.382	0.08	0.301	0.075	0.965
Education level	0.003	0.016	0.365	0.244	0.59	0.439	0.234	0.304
Parity	0.890	0.691	0.028	0.636	0.013	0.746	0.569	0.252
ANC accompaniment by spouse	0.272	0.850	0.41	0.349	0.006	0.044	0.103	0.413
PNC accompaniment by spouse	0.003	0.992	0.57	0.973	0.008	0.309	0.005	0.174
Danger signs information to mother	0.002	0.951	0.000	0.027	0.000	0.001	0.002	0.171
Explanation of MCH booklet by care provider during ANC	0.003	0.159	0.184	0.152	0.987	0.387	0.320	0.568
Mother read MCH booklet	0.002	0.951	0.000	0.027	0.000	0.001	0.984	0.212

The values in the tables are P values from Chi square tests

#### Hottness of the body as a neonatal danger signs

The mother’s level of education, having read the MCH booklet and receiving information of content of MCH booklet by the health care workers had positive influence on maternal knowledge of hottness of the body as a neonatal danger sign and this was significant with a P value less than 0.05 as shown in Table 4. Age of the mother, marital status and spousal accompaniment and parity had no influence on knowledge of postnatal neonatal danger signs.

#### Difficulty in breathing as a neonatal danger signs

Age of the mother, level of education, mothers receiving information on neonatal danger signs from care provider on neonatal danger signs was associated with increased likelihood of mothers being able to identify difficulty in breathing as a neonatal danger sign and this was significant as shown in Table 4. Marital status, partner accompaniment, reading of MCH booklet were not statistically significant factors associated with ability of the postnatal mothers to identify presence of difficulty in breathing as a neonatal danger sign.

### Multivariable regression analysis

Nine variables (inability to breastfeed, convulsion, drowsiness, difficulty in breathing jaundice, hottness of body, cold extremities and abdominal distension) were tested against the maternal knowledge. In all the factors none was statistically significant as shown in Table 5.

**Table 5:** Multivariable regression analysis of maternal factors with level of knowledge on neonatal danger signs

	df	Sig.	Odds ratio	95% confidence interval for odds ratio	
				Lower bound	Upper bound
Intercept	1	0.095			
Age	1	0.197	0.587	0.261	1.318
Marital status	1	0.843	1.102	0.422	2.874
ANC accompaniment by spouse	1	0.058**	1.819	0.981	3.375
PNC accompaniment by spouse	1	0.685	1.141	0.604	2.154
Education level	1	0.089**	0.775	0.577	1.04
Parity	1	0.225	0.699	0.392	1.247
Danger signs information to mother	1	0.057**	2.005	0.98	4.099
Mother read MCH booklet	1	0.058**	0.507	0.252	1.022
Explanation of MCH booklet by care provider	1	0.454	1.301	0.653	2.596

** Significant at 10%

## Discussion

Neonatal illnesses have been noted to contribute to high morbidity and mortality in Kenya and in the world. Efforts have been introduced to increase awareness of the danger signs among health care givers and the parents. Some Easily identifiable danger signs have been included in the mother and child booklet but the utilization of the books by both health care workers and mothers is insufficient [16, 17]. Despite the population under the study having at least basic education, most of them did not read the mother and child booklet and their knowledge of neonatal danger signs was inadequate.

In the present study the awareness of mothers in Peri- urban area of Nakuru central district regarding new born danger signs was found to be poor. Majority of the postnatal mothers had not received any information from the health care workers during the antenatal period regarding maternal and neonatal dangers signs. Maternal knowledge of neonatal danger signs was noted to be low comparable to other studies both in Africa and India [15, 18, 19]. Recognition of most neonatal danger signs by the postnatal mothers was low with 350 (84.5%) being able to identify less than three neonatal danger signs. Hottness of the body (fever) was the commonest recognized danger sign by 310 (74.9%) followed by difficulty in breathing 193 (46.6%). Other signs included poor sucking 166 (40.1%) and lethargy/unconsciousness 24 (5.8%). This is almost comparable to the study in India where, 55 (76.4%) of mothers identified fever as the new born danger sign with 29 (40.3%), 16 (22.2%) and 10 (13.9%) identified difficulty in breathing, poor sucking and lethargy/unconsciousness as new born danger signs respectively [18]. Some studies have shown lower levels of knowledge. In Ghana and Uganda it has been observed that the study participants had little knowledge regarding neonatal danger signs [14, 15]. In Ghana 93.6, 94.3, 82.5, 95.1 and 92.3% did not know that Yellow palms, baby too small, swollen abdomen, redness of umbilical stump, and unconsciousness respectively were neonatal danger signs [14]. The difference in findings in the studies could be attributed to the difference in the type of populations and the difference in level of education. In Ghana and Uganda the studies dealt with rural population with majority of people lacking basic education [14, 15] while in this study most women were from Peri-urban areas of Nakuru town who had visited a health facility and over 90% having attained some form of formal education.

There was low postnatal follow up immediately after delivery with this study showing that only 3.4% of the mothers were reported to have attended postnatal clinic within 48 h after delivery, but this could be due to failure to capture the mothers who had postnatal reviews in the delivery facility before being discharged. This differs with the KDHS rates of 51% attendance at 48 h after delivery [4]. Postnatal period has been identified as an integral duration when information is shared with the mothers. Low attendance postnatal care clinics could lead to inadequate maternal knowledge of neonatal danger signs as there is a failure of continuum of care from ANC, delivery and postnatal. Influence of level of education is similar to other studies which show that more educated women were likely to identify inability to breastfeed, hottness of the body and difficulty in breathing as postnatal danger signs [10]. More educated mothers were also likely to have read the mother child booklet and this highly influenced the maternal knowledge. Role of health care providers as source of essential health information was noted to have highly influences the maternal knowledge as the mothers who were explained the contents of the MCH booklet were likely to identify common neonatal danger signs. In this study, among the mothers at the postnatal visit, 61% reported that the content of the MCH booklet was explained to them during their antenatal visit, while 42.8% reported that neonatal danger signs were explained to them at the antenatal and this could have led to the overall low knowledge of danger signs among postnatal mothers. Healthcare providers have been identified as the source of health information and therefore inadequate message have a big impact on the overall knowledge of the postnatal mothers. Postnatal mothers who were explained the content of the MCH book were noted to be more likely to identify inability to breastfeed, hottness of the body and difficulty in breathing as neonatal danger sign compared to the mothers who had not been explained the content of the booklet. This is compared with the Palestinians’ survey that showed a high percentage of mothers who were given health information by care providers (71%) had more health information [12].

## Conclusion

Knowledge of neonatal danger signs was low among mothers attending well baby clinic despite the information being available in the Kenyan ministry of health Mother and child booklets provided during antenatal clinics by health care providers.

### Recommendation

The antenatal health care workers’ role in educating mothers on various reproductive health issues in the postnatal period should be emphasized. There is need to provide training to health care workers with emphasis on the importance of using the MCH booklet and explaining its contents to the ANC and PNC mothers. Efforts should be made to provide health messages on neonatal danger signs to all mothers attending both antenatal clinic and postnatal clinics. Adoption of the mobile telephone as a tool for delivering health information can be utilized. Supportive supervision should be provided to all health facilities in order to improve quality of maternal and neonatal health services provided in the county.

## Research design and methodology

### Study area

Nakuru central district has been re-designated as Nakuru central sub-county, it is one of the 9 sub-counties in the Nakuru County and it includes Nakuru town which is currently the fourth largest urban centre in Kenya. The county has an estimated population of 1,812,902 people. It lies about 1850 m above sea level. It is located in the Great Rift Valley and about 150 km West of Nairobi. It has a population of 473,288 people. It is a cosmopolitan district with people from different cultures. Its main economic activities are manufacturing, agriculture and tourism. Nakuru has much government, non-governmental and private health facilities. The health care in the district is provided by different levels of care which are classified as tiers with large facilities providing specialized care being in tier 4 with lowest been tier 1 in the community.

### Sampling size and sampling procedure

#### Sampling procedures for health facilities

This was purposive sampling to include government run facilities in Tier 2, 3 and 4 of health care, faith based and private facilities. The sampling frame was the 37 listed health facilities in the district which offer the package of antenatal, delivery and postnatal care. Random sampling was done from the facilities to ensure stratified representation of the facilities reflecting owning agency and tier Random sampling was done from the facilities to ensure stratified representation of the facilities reflecting owning agency and tier as follows:

- Government-run facilities
  - Provincial General Hospital, Nakuru-Tier 4
  - Annex PGH Nakuru-Tier 4
  - Lanet Health Centre-Tier 2
  - Bondeni Maternity-Tier 2.
- Faith based facilities
  - PCEA Nakuru west-Tier 2
  - Mother Kelvin-Tier 2
- Private for profit
  - Mediheal Hospital-Tier 3
  - Nakuru Nursing Home-Tier 3

#### Sampling procedure for postnatal mothers

Sample size was calculated using Fischer’s formula arriving at a sample size of 374 (Z = confidence level at 95% (standard value of 1.96), P = estimated prevalence of postnatal follow-up in Kenya 42% (KDHS 2010). 10% contingency gives a minimum sample size of 414. The data collection was conducted in Jan, Feb and March 2014. The selection of postnatal mothers was from the eight selected facilities. Postnatal mothers attending the mother and child health clinic with children aged six weeks to nine months of age were selected at random for interview by a nurse. A structured questionnaire was administered by a qualified nurse. The answers provided by the postnatal mothers were filled in the questionnaire by the nurse. The questionnaires were filled daily in the government facilities because well baby clinic are carried out on daily basis but it was done on specific days in other facilities where MCH clinics are performed once weekly.

### Data management

The processing of data involved office editing, coding of open-ended questions, data entry, and editing inconsistencies found by computer programmes. Data was processed using the SPSS software (version 20).

## Abbreviations

ANC: antenatal care
IMNCI: Integrated Management of Newborn and Childhood Illness
KDHS: Kenya demographic health survey
LICS: low income countries
MCH: mother and child health
SPSS: Statistical Package for Social Science
WHO: World Health Organization
PNC: postnatal care
